# Biopsychosocial correlates of psychoactive substance use among student athletes in Azerbaijan

**DOI:** 10.1007/s44192-026-00392-w

**Published:** 2026-02-14

**Authors:** Aynur Bunyatova, Tahmina Taghi-zada, Lala Ahmadova, Lala Mursalbayova, Arzu Suleymanova

**Affiliations:** 1 Azerbaijan Sports Academy, Baku, Azerbaijan; 2 Azerbaijan National Antidoping Agency (AMADA), Baku, Azerbaijan; 3 Azerbaijan State Pedagogical University, Baku, Azerbaijan

**Keywords:** Psychoactive substance use, Student-athletes, Biopsychosocial model, Emotional exhaustion, Psychosocial stress, Azerbaijan, Cross-sectional study

## Abstract

**Background:**

Student-athletes in transitional post-Soviet societies may experience elevated psychosocial strain across academic, athletic, and socio-cultural domains, which may be associated with psychoactive substance (PAS) use. Empirical evidence describing PAS involvement within an integrated biopsychosocial framework in this population remains limited.

**Methods:**

A cross-sectional survey was conducted among 1,503 student-athletes aged 17–25 years at the Azerbaijan Sports Academy (ASA). PAS-related characteristics were assessed using a newly developed, exploratory four-factor PAS Risk Questionnaire capturing social environment, psychological vulnerability, substance-use behaviours, and biopsychosocial stressors (internal consistency α = 0.74–0.85). Data were analysed using descriptive statistics, Pearson correlation analyses, independent-samples t-tests, one-way ANOVA, and mediation analysis (PROCESS Model 4).

**Results:**

Substance-use behaviour scores showed modest-to-moderate positive associations with emotional exhaustion (*r* = .44), perceived stress (*r* = .33), and adverse social-context indicators such as family conflict (*r* = .37), all *p* < .001. Male athletes and older students (21–25 years) reported higher substance-use behaviour scores compared with their counterparts (*p* < .01). Mediation analysis suggested an indirect association pattern in which higher academic stress corresponded to higher substance-use behaviour scores through emotional exhaustion (indirect effect, 95% CI [0.11, 0.22]).

**Conclusions:**

PAS use among student-athletes in this context appears to co-occur with interconnected psychological, social, and physical stress-related characteristics, rather than representing isolated behaviours. These preliminary, exploratory findings highlight the potential importance of culturally responsive mental health and preventive strategies within sport-education systems. Given the cross-sectional design and the exploratory, non-validated nature of the PAS Risk Questionnaire, causal interpretations and generalization beyond this context should be made with caution. Further longitudinal research and independent psychometric validation of the instrument are warranted.

**Supplementary Information:**

The online version contains supplementary material available at 10.1007/s44192-026-00392-w.

## Introduction

Psychoactive substance (PAS) use among young people represents a growing global public health concern and a major challenge to achieving Sustainable Development Goal 3: Good Health and Well-being [19]. Substance use typically emerges during late adolescence and early adulthood—a developmental period marked by identity exploration, increasing autonomy, reduced parental monitoring, and heightened exposure to social and academic demands [1, 14]. Contemporary research further indicates that transitions to adulthood have become increasingly prolonged and fragmented, intensifying cumulative academic, social, and identity-related stressors during this life stage [15]. International monitoring data indicate that the average age of first PAS use ranges between 16 and 17 years in most European countries [6] and approximately 16.5–17.5 years in the United States [9]. In Azerbaijan, however, national reports suggest an earlier initiation, often around 14–15 years of age (State Committee on Family, [17]), highlighting the importance of examining psychosocial strain and patterns of early psychoactive substance exposure during the transition into higher education.

In parallel, the mental health of athletes has increasingly been recognized as a global public health priority [12], [21]. Although athletic participation is often assumed to confer psychological resilience, university student-athletes face distinctive stressors related to performance expectations, academic–sport balance, competitive evaluation, and injury risk. Empirical evidence indicates that symptoms of burnout, anxiety, sleep disturbances, and emotional exhaustion among athletes occur at rates comparable to—or exceeding—those observed in non-athlete student populations [4, 7]. Importantly, psychological distress in sport contexts may be accompanied by coping-related behavioural patterns, including engagement with psychoactive substances [20]. Chronic psychosocial stress has been shown to disrupt affective regulation and coping mechanisms, which may coincide with higher levels of substance-use behaviours under conditions of prolonged emotional strain [16].

International research further indicates that sport participation does not uniformly protect against psychoactive substance (PAS) use. National data from U.S. collegiate athletes reveal that male NCAA athletes who use performance-enhancing substances are significantly more likely to engage in broader substance use, including alcohol, marijuana, and other illicit substances [2]. Longitudinal evidence also suggests that participation in organized team sports—distinct from general physical activity—is associated with higher consumption of alcohol and smokeless tobacco [18].

Environmental and policy analyses from the Canadian Centre on Substance Use and Addiction [3] further demonstrate that sport involvement may function as both a protective and a risk-enhancing context, depending on team norms, competitive pressure, and institutional culture. These findings underscore the importance of examining substance-related vulnerability among student-athletes within specific psychosocial and organizational environments, rather than assuming a uniformly protective role of sport participation.

While substantial progress has been made in Western countries through school-based prevention, anti-doping education, and integrated mental health services, comparable support structures remain under-developed across much of the South Caucasus. Institutional constraints, limited availability of psychological services, persistent stigma surrounding mental health care, and dominant masculine norms—particularly within sport environments—may restrict adaptive coping and help-seeking behaviors. Regional epidemiological studies indicate that substance use and PAS exposure remain prevalent among adolescents and young adults in post-Soviet contexts. For example, school-based surveys in Georgia have documented measurable levels of alcohol, tobacco, and illicit drug use among adolescents [10], while broader analyses of post-Soviet regions highlight structural gaps in public health responses, treatment access, and harm-reduction infrastructure [22]. These findings suggest that prevention frameworks developed in Western contexts cannot be directly transferred to the region without cultural and institutional adaptation.

Although the student-athletes examined in this study were born after the dissolution of the Soviet Union, their educational and athletic environments continue to be shaped by post-Soviet institutional heritage. In this context, the term *post-Soviet educational system* refers not to participants’ personal experience of Soviet schooling, but to the persistence of institutional practices, organizational norms, and cultural expectations inherited from the Soviet sport and education model. These include hierarchical coach–athlete relationships, strong emphasis on physical discipline and performance outcomes, limited institutional attention to psychological well-being, and residual stigmatization of help-seeking for emotional difficulties. Post-independence reforms have introduced greater internationalization and competitiveness within sport education; however, these changes have often occurred without parallel expansion of mental health infrastructure, potentially increasing psychosocial strain among student-athletes.

Recent research further suggests that individual differences and generational characteristics may interact with these institutional conditions. Personality-related tendencies toward emotional suppression, risk-taking, and stress-reactive coping have been documented among athlete populations [11]. In parallel, studies focusing on Generation Z highlight heightened sensitivity to uncertainty, performance evaluation, and social comparison within sport environments [8]. Although these factors were not directly measured in the present study, they may function as important contextual influences shaping vulnerability to PAS involvement and therefore warrant consideration in the interpretation of the findings.

The Azerbaijani sociocultural context also plays a significant role in shaping coping strategies and help-seeking behaviors. Strong collectivist family structures, respect for authority, and culturally embedded expectations of emotional restraint—particularly among men—may foster discipline and perseverance while simultaneously discouraging open discussion of psychological distress. These norms are reflected in national folklore and oral traditions, most notably in the epic «*Dada Qorqud»*, where heroic figures embody ideals of endurance, loyalty, and silent perseverance in the face of adversity. While such narratives may cultivate resilience and commitment—qualities valued in athletic settings—they may also normalize internalization of stress and limit adaptive emotional expression when academic or performance pressures accumulate. At the same time, Azerbaijani culture offers a range of culturally embedded protective resources that may support emotional regulation, social cohesion, and adaptive coping. These include strong traditions of communal solidarity, nationally shared rituals and celebrations (such as Nowruz), music, and a rich heritage of traditional games and physical practices. Historical and cultural forms of collective activity—such as *«chovgan», «zorkhana practices», «pehlivan wrestling», «chalagan chardagy»,* and other traditional games—have long functioned as mechanisms for social bonding, identity formation, and regulation of emotional and physical strain. Despite their potential relevance for psychosocial well-being, such indigenous cultural and physical practices remain largely underutilized in formal mental health promotion and psychoactive substance–use prevention programs.

Existing international evidence demonstrates that psychosocial factors such as family conflict, emotional dysregulation, chronic stress, and permissive social norms are consistently associated with PAS involvement among youth [13]. However, the operation of these mechanisms within culturally distinct, transitional educational systems—particularly among student-athletes—remains insufficiently explored. This gap underscores the need for empirically grounded, context-sensitive research that integrates psychological, social, and institutional dimensions of risk.

Against this background, the present study applies Engel’s [5] biopsychosocial model as an integrative framework for examining PAS involvement among student-athletes in a post-Soviet academic setting. The model conceptualizes substance-related behavioural tendencies as emerging within the interaction of emotional strain (e.g., stress and exhaustion), social context (e.g., family and peer environments), and biological or physiological demands associated with intensive training and academic workload. By situating PAS involvement within this multidimensional framework, the study challenges assumptions that athletic participation is inherently protective and highlights the importance of culturally responsive prevention strategies in sport-education systems. Accordingly, this study investigates psychoactive substance involvement and substance-related behavioural tendencies among student-athletes enrolled in a post-Soviet higher-education institution. Based on the biopsychosocial framework and existing empirical evidence, the following hypotheses were tested:

**H1.** Higher levels of emotional distress and perceived stress will be positively associated with self-reported psychoactive substance use among student-athletes.

**H2.** Higher levels of physical fatigue and emotional exhaustion will be associated with higher substance-use behaviour scores.

**H3.** Student-athletes aged 21–25 will report higher substance-use behaviour scores compared with those aged 17–20.

## Methods

This study did not involve a clinical trial and was therefore not subject to mandatory registration. Data were collected via an anonymous online questionnaire related to sport psychology and mental health. No medical or pharmacological interventions were used, and all participants provided informed consent.

### Study design and participants

A cross-sectional online survey was conducted in November 2024 among undergraduate and graduate students enrolled at the Azerbaijan Sports Academy (ASA). The target population included all actively enrolled students across academic programmes in sport sciences, coaching, sport management, and physical rehabilitation. According to institutional reports, ASA educates approximately 7000 students annually, providing the estimated size of the reference population for this study.

Survey invitations were distributed through official academic channels coordinated by the Dean’s Office, including faculty advisors, programme tutors, and course group coordinators via institutional e-mail lists and internal student communication platforms. In addition, brief in-class announcements were made to inform students about the study and provide access to the survey link. No data collection took place in classrooms, and instructors were not present during survey completion to avoid coercion or perceived pressure to participate.

Participation was voluntary, and the sample therefore represents a self-selected convenience sample, which is typical for institution-based online surveys. Based on the estimated population size (~ 7,000 students), the final sample of N = 1,503 corresponds to an approximate response rate of 21–22%. This recruitment method may introduce a degree of self-selection bias, as students with higher interest or personal relevance to the topic may have been more likely to participate; this consideration is addressed further in the study limitations.

Inclusion criteria were: (a) active enrolment at ASA; (b) age between 17 and 25 years; and (c) ability to provide informed consent. Eligibility was enforced through automatic filters embedded in the online questionnaire.

A total of N = 1,503 students completed the questionnaire. Gender was reported by 1,480 participants: 78.99% male (n = 1,187) and 19.51% female (n = 293). A small proportion (1.53%, n = 23) did not report gender and were retained only in analyses that did not require gender information. Demographic characteristics and contextual indicators of PAS exposure are presented in Table 1**.**

**Table 1:** Demographic characteristics and contextual indicators of PAS exposure (N = 1503)

Variable	Category	N	%
Demographic characteristics			
Age group	17–20 years	1,366	90.9
	21–25 years	137	9.1
Gender	Male	1,187	78.99
	Female	293	19.51
	Not reported / missing	23	1.53
PAS exposure indicators			
PAS exposure frequency^1^	1–2 times per month	466	31.01
	Daily	220	14.65
Contextual triggers of PAS exposure^1^			
Situational triggers	Academic stress	701	46.61
	Social situations	652	43.39
Psychosocial contexts related to PAS exposure^1^			
Psychosocial contexts	Interpersonal conflict	533	35.46
	Uncertainty about future	524	34.84
	Financial difficulties	199	13.21
	Academic pressure	134	8.90

Percentages for PAS exposure indicators, situational triggers, and psychosocial contexts represent endorsement rates; multiple responses were permitted, and percentages do not sum to 100%. Reported categories refer to the frequency of exposure to PAS-related environments rather than to actual substance consumption. PAS exposure indicates being present in contexts where psychoactive substances are available and does not imply self-reported PAS use.

### Survey instrument and conceptual framework

The survey instrument was developed within a biopsychosocial conceptual framework to assess multiple vulnerability pathways associated with psychoactive substance (PAS) involvement among student-athletes.

Following pilot testing and minor item refinement aimed at improving clarity and cultural relevance, the final version of the questionnaire consisted of 22 items organised into four conceptually distinct subscales. No substantive changes to the conceptual structure of the instrument were introduced during this process.

The structural composition of the PAS Risk Questionnaire is presented in Table 2**.**

**Table 2:** Structure of the PAS risk questionnaire subscales

Subscale	Example content	Items
Psychological vulnerabilities	perceived stress, emotional exhaustion, anxiety, coping difficulties	6
Social environment	peer influence, availability and accessibility of PAS	5
Substance-use behaviours	frequency and situational context of PAS use	5
Biopsychosocial stressors	physical fatigue, academic overload, craving	6

Although craving may conceptually overlap with substance-use tendencies, it was classified as a biopsychosocial stressor in the present study. This decision reflects student-athletes’ reports of craving-like urges emerging in response to academic overload and cumulative physical strain, even in the absence of actual PAS consumption, thereby preserving conceptual distinction between psychosocial vulnerability and behavioural engagement.

All items were rated on a 5-point Likert scale (1 = *Never* to 5 = *Very often*). Negatively worded items were reverse-coded prior to computing subscale scores. Higher scores indicated greater psychosocial vulnerability to PAS involvement.

To avoid conceptual overlap and inflation of associations, behavioural items (Substance-use behaviours**)** were analytically separated from psychosocial risk indicators. Behavioural items were not used as predictors in inferential models and were treated exclusively as outcome-related measures.

In addition to the subscales above, selected items assessed PAS exposure (presence in environments where substances were available or used) and PAS use (self-reported consumption frequency). These constructs were conceptually and analytically distinguished, as described below.

Example items included:

Psychological vulnerability: “I experience emotional exhaustion due to academic and sport-related demands.”

Social environment: “People close to me are frequently exposed to psychoactive substances.”

Substance-use behaviours: “I use psychoactive substances in response to stress or emotional pressure.”

Biopsychosocial stressors: “Academic workload and training demands cause significant physical fatigue.”

### PAS outcomes: exposure, use, and composite risk score

Three complementary indicators were operationalised to distinguish contextual availability, behavioural engagement, and overall psychosocial vulnerability to PAS involvement. In addition, academic stress was operationalized as the mean score of the academic overload items included in the Biopsychosocial Stressors subscale and used as the predictor variable in mediation analyses**.**

PAS exposure (contextual availability indicator)

- Definition: frequency of presence in environments where PAS were available or consumed
- Items: 2 situational items referring to different exposure contexts (e.g., social or institutional settings)
- Response scale: 1 = *Never* to 5 = *Very often*
- Score calculation: mean of items
- Internal consistency: α = 0.78
- Analytical role: descriptive only

Percentages reported in Table 1 reflect endorsement rates across multiple exposure contexts rather than mutually exclusive frequency categories.

PAS use (primary behavioural outcome)

- Definition: self-reported frequency of PAS consumption
- Items: 3 behaviour-oriented items
- Response scale: 1 = *Never* to 5 = *Very often*
- Score calculation**:** mean of items
- Internal consistency: α = 0.82
- Analytical role**:** primary dependent variable in inferential analyses
- Reported in results as: *PAS involvement (self-reported use)*

PAS risk composite (descriptive biopsychosocial vulnerability index)

- Definition: aggregated index reflecting overall psychosocial vulnerability to PAS involvement
- Components: all four subscales (22 items total)
- Score calculation: mean total score
- Internal consistency**:** α = 0.81
- Analytical role: descriptive index only

The PAS risk composite was not used as a predictor in inferential statistical models. The PAS risk composite was not entered as a predictor in inferential models in order to avoid conceptual overlap with behavioural outcome indicators. The measurement characteristics and descriptive statistics for all three indicators are presented in Table 3.

**Table 3:** PAS outcomes: measurement characteristics and descriptive statistics (N = 1,503)

Variable	Items	Scale	α	M	SD	Analytical role
PAS exposure	2	1–5	0.78	2.41	0.86	Contextual exposure indicator
PAS use (self-reported)	3	1–5	0.82	1.68	0.91	Primary behavioural outcome
PAS risk composite	22	1–5 (mean score)	0.81	2.30	0.64	Composite psychosocial vulnerability index

PAS exposure reflects endorsement across multiple situational contexts rather than mutually exclusive frequency categories.

### Instrument validation and pilot testing

A pilot test (n = 45) was conducted prior to the main data collection to evaluate item clarity, linguistic adequacy, and cultural relevance for Azerbaijani student-athletes. Based on participants’ feedback and expert review, minor wording refinements were introduced (for example, clarifying examples of academic stress and training load). No items were added or removed; the final instrument retained 22 items across the four planned subscales.

In the full sample (N = 1503), internal consistency was acceptable to excellent across all subscales, with Cronbach’s α values ranging from 0.74 to 0.85. The total scale also demonstrated high internal reliability (α = 0.81). Internal consistency coefficients for each subscale and the full instrument are presented in Table 4.

**Table 4:** Internal consistency coefficients (Cronbach’s alpha) for PAS risk questionnaire subscales

Subscale	Conceptual domain	Number of items	Cronbach’s α (95% CI)
Social environment	Peer influence and institutional support	5	0.74 [0.71–0.77]
Psychological vulnerabilities	Emotional distress, anxiety, coping difficulty	6	0.82 [0.79–0.84]
Substance-use behaviours	Frequency and situational context of PAS use	5	0.77 [0.74–0.80]
Biopsychosocial stressors	Academic overload, physical fatigue, craving	6	0.85 [0.83–0.87]
Full scale	Total instrument	22	0.81 [0.79–0.83]

Exploratory factor analysis (EFA) was conducted using principal axis factoring with an oblique rotation (Promax), reflecting the theoretical expectation that biopsychosocial domains are moderately interrelated. Sampling adequacy was strong (Kaiser–Meyer–Olkin = 0.89), and Bartlett’s test of sphericity was significant (*p* < 0.001), indicating suitability for factor analysis.

The number of factors was determined based on eigenvalues greater than 1, inspection of the scree plot, and theoretical interpretability. Four interpretable factors emerged, corresponding to the predefined subscales, and together explained approximately 63% of the total variance. Standardised primary factor loadings were generally moderate to high, with low cross-loadings and communalities exceeding 0.40, supporting a coherent multidimensional structure of PAS-related vulnerability. Detailed factor loadings and communalities are presented in Supplementary Table 1.

Although the four-factor structure was theoretically specified a priori, exploratory rather than confirmatory factor analysis was considered appropriate given the newly composed and culturally adapted nature of the instrument and the absence of prior psychometric validation in post-Soviet sport-education settings. This approach allowed for empirical examination of the latent structure before confirmatory testing. As EFA and substantive analyses were conducted on the same sample, results should be interpreted with caution due to potential capitalization on chance. Future research should replicate these findings using confirmatory factor analysis in independent samples to formally test model fit and further validate the proposed biopsychosocial model.

The internal consistency estimates closely aligned with the factor analytic results, with higher Cronbach’s α values corresponding to stronger and more homogeneous factor loadings across all four subscales.

### Data collection procedure

The survey was administered online via Microsoft Forms in November 2024. Prior to accessing the questionnaire, participants received a brief description of the study and provided electronic informed consent. Participation was voluntary, anonymous, and had no academic consequences. Students completed the survey individually at a time and place of their choosing. No personally identifying information (e.g., names, email addresses, IP addresses) was collected.

Data quality screening procedures were implemented to minimise inattentive or satisficing responses. Responses exhibiting implausibly short completion times, patterned responding, or more than 20% missing data were excluded from the final dataset (n = 14). Cases with minimal missingness (< 5%) were retained. Missing responses within subscales were handled using person-mean substitution, a commonly applied approach in scale-based research when missingness is low and items demonstrate adequate internal consistency. This strategy was chosen to preserve statistical power while minimising bias.

The study protocol complied with institutional ethical standards and received approval from the Ethics Committee of the Azerbaijan Sports Academy (Protocol No. 13, dated 03/11/2024). Participants were informed of their right to withdraw from the study at any time without consequences.

### Statistical analysis

Statistical analyses were conducted using IBM SPSS Statistics 25. Composite scores derived from Likert-type items were treated as continuous variables, which is considered appropriate in large samples with approximately normal distributions. Although individual Likert-type items are ordinal, treating aggregated scale scores as continuous is consistent with established methodological recommendations for behavioural research.

Analyses comprised descriptive statistics, Pearson correlation analyses, independent-samples *t*-tests, and one-way analyses of variance (ANOVA). Effect sizes were reported as Cohen’s *d* for *t*-tests and η^2^ for ANOVA to facilitate interpretation of practical significance.

Mediation analysis was conducted using PROCESS Model 4 with 5,000 bootstrap samples (PROCESS macro v4.2 for SPSS). Given the cross-sectional design, mediation results are interpreted strictly as statistical associations consistent with the proposed model, rather than as evidence of temporal or causal relationships.

As a diagnostic check for potential common method bias associated with self-report measures, Harman’s single-factor test was conducted. The first unrotated factor accounted for less than 40% of the total variance, suggesting that common method variance was unlikely to constitute a dominant source of bias. The limitations of this diagnostic approach are acknowledged in the Limitations section.

Assumptions of normality (skewness, kurtosis, and Q–Q plots), homogeneity of variance (Levene’s tests), and independence of residuals were examined prior to inferential analyses; no substantial violations were detected.

An a priori power analysis indicated that the sample size provided sufficient power (> 0.80) to detect small-to-moderate effects (f^2^ ≈ 0.05). No formal correction for multiple comparisons was applied, as the analyses were primarily exploratory and theory-driven; therefore, findings should be interpreted with appropriate caution regarding Type I error.

## Results

### Descriptive statistics

Descriptive characteristics of the sample are presented in Table 1. PAS exposure was reported at least monthly by 31.01% of respondents, while 14.65% endorsed daily exposure contexts. Academic stress (46.61%) and social situations (43.39%) were the most frequently reported PAS-related exposure contexts.

Endorsed psychosocial vulnerability contexts were most frequently related to interpersonal conflict (35.46%) and uncertainty about the future (34.84%), whereas financial difficulties were less frequently reported (13.21%). Overall, these descriptive patterns indicate a higher frequency of psychosocial and emotional contexts compared with economic strain within PAS-related exposure and risk indicators in this population.

### Correlation analysis

Pearson product–moment correlations were conducted to examine associations between psychosocial risk domains and PAS use (self-reported). Higher scores reflected higher psychosocial risk or more frequent substance-use behaviour. Given the large sample size, all observed correlations reached statistical significance at p < 0.001. Means, standard deviations, and correlation coefficients are presented in Table 5**.**

**Table 5:** Means, standard deviations, and correlations among PAS risk domains and PAS use (Self-Reported) (N = 1,503)

Variable	M	SD	1	2	3	4	5
1. Psychological vulnerabilities	2.38	0.67	–	0.47***	0.39***	0.33***	0.44***
2. Social environment	2.41	0.61	0.47***	–	0.42***	0.29***	0.37***
3. Biopsychosocial stressors	2.33	0.65	0.39***	0.42***	–	0.35***	0.41***
4. Substance-use behaviours	2.26	0.72	0.33***	0.29***	0.35***	–	0.52***
5. PAS use (self-reported)	1.68	0.91	0.44***	0.37***	0.41***	0.52***	–

Pearson correlation coefficients are reported. **p < 0.001

As shown in Table 5, the strongest association was observed between substance-use behaviours and PAS use (self-reported) (*r* = 0.52, *p* < 0.001), reflecting their conceptual proximity as behavioural indicators. Psychological vulnerabilities also demonstrated a robust positive association with PAS use (*r* = 0.44, *p* < 0.001), showing that emotional exhaustion, perceived stress, and coping difficulties were among the psychosocial variables most strongly correlated with PAS use (self-reported).

Moderate positive associations were likewise observed between PAS use and both biopsychosocial stressors (*r* = 0.41, *p* < 0.001) and social environment factors (*r* = 0.37, *p* < 0.001), with higher PAS use scores observed alongside cumulative academic demands, physical fatigue, and stressful social-context indicators. Taken together, these findings support a multidimensional pattern of psychosocial correlates consistent with a biopsychosocial conceptualization of PAS-related involvement and associated psychosocial characteristics.

### Group comparisons

Group differences in substance-use behaviours, reflecting the frequency and situational context of psychoactive substance–related behaviours, were examined by gender**,** age group, and levels of emotional exhaustion (tertiles). This behavioural indicator was analysed separately from PAS use (self-reported consumption) to maintain conceptual clarity between behavioural tendencies and actual substance use**.**

Assumptions of homogeneity of variance were met for all comparisons (Levene’s tests, *p* > 0.05). Descriptive statistics, inferential test results, and effect sizes are presented in Table 6.

**Table 6:** Group differences in substance-use behaviours (N = 1503)

Comparison	Group	M	SD	Test statistic	p	Effect size
Gender	Male	2.41	0.78	*t*(1478) = 2.81	0.005	*d* = 0.18
	Female	2.18	0.73			
Age group	21–25 years	2.45	0.83	*t*(1501) = 2.45	0.014	*d* = 0.22
	17–20 years	2.29	0.76			
Emotional exhaustion (tertiles)	Low	2.21	0.71	*F*(2, 1499) = 6.72	0.001	η^2^ = 0.009
	Moderate	2.31	0.74			
	High	2.47	0.79			

Higher scores indicate more frequent and contextually embedded substance-use behaviours.

Emotional exhaustion tertiles were derived from the Psychological Vulnerabilities subscale and were analytically distinct from the physical fatigue indicators included in the Biopsychosocial Stressors subscale.

Participants with missing gender data were excluded listwise from gender comparisons (valid n = 1480). d = Cohen’s d; η^2^ = partial eta squared.

Male students reported significantly higher substance-use behaviour scores than female students,

$$t\left( {1478} \right)\, = \,2.81,p\, = \,0.005,{\text{ Cohen}}\prime {\mathrm{sd}}\, = \,0.18.$$

Students aged 21–25 years demonstrated higher substance-use behaviour scores compared to those aged 17–20 years,

$$t\left( {{15}0{1}} \right)\, = \,{2}.{45},p\, = \,0.0{14},d\, = \,0.{22}.$$

A one-way analysis of variance revealed a significant effect of emotional exhaustion level on substance-use behaviours,

$$F\left( {{2},{ 1499}} \right)\, = \,{6}.{72},p\, = \,0.00{1},{\text{ partial}}\eta^{{2}} \, = \,0.00{9}.$$

Post hoc Tukey tests indicated that students in the high emotional exhaustion group reported significantly higher substance-use behaviour scores than those in the low exhaustion group (*p* = 0.002). Differences involving the moderate exhaustion group were not statistically significant.

Overall, effect sizes across all comparisons were small, reflecting modest magnitude of observed group differences. Although statistically significant, these findings are consistent with multifactorial models of behavioural risk in large samples and should be interpreted in terms of practical rather than deterministic significance.

### Mediation analysis

A mediation analysis using PROCESS Model 4 revealed a statistically significant **indirect association pattern** between academic stress and substance-use behaviour scores via emotional exhaustion. The indirect effect was supported by bootstrapped confidence intervals that did not include zero, indicating a pattern **consistent with an indirect effect structure within this statistical model**. Given the cross-sectional design of the study, this finding should be interpreted as a statistical association rather than evidence of a causal mechanism. Standardized estimates for the total, direct, and indirect effects are presented in Table 7**.**

**Table 7:** Mediation effects of emotional exhaustion on the association between academic stress and substance-use behaviour scores (N = 1503)

Effect	β	SE	p	95% CI (LL–UL)
Total effect	0.37	0.04	< 0.001	–
Direct effect	0.21	0.04	< 0.01	–
Indirect effect (via emotional exhaustion)	0.16	–	–	[0.11, 0.22]

All coefficients are standardized. Bootstrap confidence intervals are based on 5000 resamples

The bootstrap confidence interval for the indirect effect did not include zero, indicating a statistically significant indirect association between academic stress and substance-use behaviour scores via emotional exhaustion. At the same time, the direct effect remained significant, indicating a pattern consistent with partial statistical mediation.

The overall model accounted for 29% of the variance in substance-use behaviour scores (R^2^ = 0.29). These findings indicate that emotional exhaustion is a psychological variable statistically associated with both academic stress and substance-use behaviour scores within this analytical framework. However, due to the cross-sectional nature of the data, no conclusions regarding temporal ordering or causal pathways can be drawn.

## Discussion

This study examined psychoactive substance (PAS) use and substance-related behavioural tendencies among student-athletes in a transitional post-Soviet educational context using a biopsychosocial framework. The findings provide preliminary evidence that substance-use behaviour scores are statistically associated with academic stress, emotional exhaustion, physical fatigue, and social-environment indicators. Taken together, these patterns suggest that substance-related behaviours co-occur with interrelated psychological, biological, and sociocultural stress processes, rather than reflecting isolated individual factors.

The PAS Risk Questionnaire was developed for exploratory research and demonstrated acceptable internal consistency in this sample, providing meaningful insights into substance-use behaviours among student-athletes. Accordingly, these results should be interpreted as hypothesis-generating rather than confirmatory evidence.

Consistent with international research on athlete well-being (e.g., [4, 7]), psychological vulnerabilities—particularly emotional exhaustion—showed the strongest associations with substance-use behaviour scores, indicating that depletion of emotional and cognitive coping resources is statistically associated with more frequent substance-related tendencies, without implying causal direction.

The mediation analysis revealed a statistical association suggesting that academic stress and substance-use behaviour scores are indirectly linked through emotional exhaustion. This pattern highlights a potential pathway connecting academic stress, emotional exhaustion, and substance-use behaviours, warranting further investigation in future studies using validated instruments, independent samples, and longitudinal designs. Emotional exhaustion may represent one of several interrelated psychological conditions associated with these behaviours, rather than a definitive explanatory mechanism.

Physical fatigue also showed a small but statistically significant association with PAS use. Fatigue and emotional exhaustion were treated as conceptually related but analytically distinct constructs, reflecting physical load and psychological strain, respectively. While sport participation is often assumed protective, these findings align with evidence suggesting that high training demands and insufficient recovery may co-occur with substance-related behavioural tendencies, particularly when combined with academic pressures and limited psychosocial support.

Gender- and age-related differences were observed in substance-use behaviour scores, although effect sizes were small. Male athletes reported higher scores than female athletes, possibly reflecting culturally embedded masculine norms in Azerbaijan emphasizing toughness, emotional restraint, and self-reliance. Older students (21–25 years) also showed higher scores compared with younger peers, which may reflect differences in autonomy, institutional supervision, and cumulative exposure to academic, athletic, and social demands.

Unmeasured dispositional characteristics (e.g., impulsivity, emotional suppression, stress-reactive coping styles) and Generation Z–specific contextual factors may constitute important confounders. Athletes’ personality traits—such as emotional inhibition, competitiveness, and risk-taking tendencies—have been linked to behavioural responses under psychosocial strain [11], and emerging research highlights sensitivity to digital social comparison and performance evaluation [8]. Although not assessed here, these factors should be incorporated in future research on athlete mental health and PAS involvement.

The observed psychosocial patterns should be interpreted within the cultural and institutional context of Azerbaijan. Culturally embedded norms emphasizing perseverance, emotional restraint, and collective responsibility—particularly in male-dominated sport environments—may influence how academic stress and emotional exhaustion are experienced and managed. Protective cultural resources, such as communal solidarity, shared rituals (e.g., Nowruz), music, storytelling, and traditional physical practices, may support emotional regulation and social cohesion. These contextual influences are not causal explanations of PAS involvement but underscore the importance of culturally responsive prevention and mental health strategies.

Although associations were modest, they provide initial insights that may inform early identification of psychosocial strain and targeted support in sports-education contexts. These interpretations must be considered preliminary, given the exploratory nature of the data, the cross-sectional design, reliance on self-report measures, and the single-institution sample.

In summary, this study contributes preliminary, exploratory evidence regarding the biopsychosocial correlates of PAS involvement among student-athletes in a transitional educational context. All findings should be interpreted cautiously, and future research should employ validated instruments, independent samples, and longitudinal designs to clarify the associations observed here.

## Practical implications

Given the exploratory and context-specific nature of the present findings, the following practice-oriented directions should be considered tentative suggestions for future research, policy development, and interventions in sport-education environments, rather than prescriptive or universally applicable recommendations:

- **Integrated mental health education**
– Incorporate stress management, emotional regulation, and anti-doping literacy within sport-education curricula to support student-athletes’ psychosocial well-being.
- **Gender-sensitive and stigma-free psychological services**
– Develop counselling services with clinician training focused on addressing masculinity-related barriers to help-seeking, particularly in male-dominated sport contexts.
- **Prevention through recovery and workload monitoring**
– Systematically monitor training load, fatigue, and sleep patterns to promote recovery-oriented sport cultures and reduce potential psychosocial strain.
- **Family and peer involvement**
– Conduct awareness initiatives targeting family and peer networks to address culturally embedded norms related to toughness, shame, and performance identity, which may influence coping strategies and help-seeking behaviours.
- **Digital and peer-led interventions**
– Use online psychoeducation platforms and peer-support formats to provide accessible, low-threshold communication channels for student-athletes, complementing institutionally embedded support systems.

Taken together, these directions highlight the potential utility of biopsychosocially informed, culturally responsive, and contextually tailored interventions in sport-education settings. Given the preliminary nature of the data and the use of a non-validated instrument, these recommendations should be interpreted with caution and further evaluated in longitudinal, multi-institutional, and cross-cultural studies before broader implementation.

## Limitations

Several limitations should be acknowledged:

- **Self-report measures:** All variables (predictors, mediator, and outcomes) were assessed via self-report in a single online session, introducing potential common method bias and social desirability effects, particularly given the sensitive nature of PAS use and cultural stigma. Harman’s single-factor test indicated that method bias was unlikely to dominate, but future studies would benefit from multi-method designs, temporal separation of measurements, or analytic approaches modeling method variance.
- **Cross-sectional design:** The cross-sectional nature of the study precludes causal inference. Observed associations, including those suggested by the mediation analysis, should be interpreted as exploratory and hypothesis-generating, rather than indicative of temporal or causal mechanisms.
- **Limited covariate adjustment:** The mediation model did not include additional covariates (e.g., gender, age, physical fatigue, social support), which may influence observed associations. Future research should replicate findings using longitudinal designs and extended models with theoretically relevant covariates.
- **Instrument limitations:** The PAS Risk Questionnaire showed internal consistency, but exploratory factor analysis was conducted on the same dataset used for substantive analyses. The instrument has not been independently validated, increasing the risk of capitalization on chance. Future studies should employ independent samples and confirmatory factor analysis (CFA) to formally validate the instrument.
- **Sample representativeness:** Participants were recruited from a single sport-education institution, which may introduce self-selection bias and limit generalizability to other universities, non-athlete populations, or different cultural contexts. Socioeconomic status and type of sport were not assessed, further constraining interpretation.
- **Gender imbalance:** The sample was predominantly male, reflecting institutional composition. This limits interpretation of gender comparisons and generalizability to gender-balanced sport-education settings.

Taken together, these methodological, instrument-related, and contextual limitations highlight the need for longitudinal, multi-institutional, and cross-cultural research incorporating broader psychological, social, and contextual indicators. All findings should be interpreted as preliminary and hypothesis-generating, providing insights to guide future research rather than definitive conclusions.

## Conclusion

This study contributes to the literature on athlete mental health by examining psychoactive substance–related patterns among student-athletes through a biopsychosocial lens in an underrepresented post-Soviet educational context. The findings suggest that sport participation alone is not uniformly associated with lower levels of substance-related behaviours. Instead, higher levels of emotional exhaustion, academic stress, challenging social environments, and physical fatigue were statistically associated with substance-use behaviours and self-reported PAS use.

These results are consistent with the potential relevance of integrated mental health approaches within sport-education systems. Preventive strategies may benefit from prioritising early identification of psychosocial strain, recovery-oriented training practices, and access to stigma-sensitive and gender-aware support services. In culturally specific contexts such as Azerbaijan, mental health promotion may also be strengthened by incorporating culturally informed and collectively oriented forms of outreach that align with local values, traditions, and social norms.

Although these findings are context-specific and derived from cross-sectional, exploratory data using a non-validated instrument, the observed associations are broadly consistent with psychosocial patterns documented across athlete populations internationally. Future research employing validated measures, independent samples, and longitudinal or cross-cultural designs will be important to clarify developmental trajectories, temporal ordering, and contextual moderators of substance-related involvement. Such work may inform the development of holistic, person-centred sport education models that balance performance demands with psychological well-being and align with global public health priorities, including Sustainable Development Goal 3: Good Health and Well-being.

## Supplementary Information

Below is the link to the electronic supplementary material.

Supplementary Material 1

## Data Availability

The datasets generated and/or analysed during the current study are available from the corresponding author upon reasonable request.
