# Supplementary material for: Biopsychosocial correlates of psychoactive substance use among student athletes in Azerbaijan
Source: Discov Ment Health. 2026 Feb 14;6(1):42. doi: 10.1007/s44192-026-00392-w (PMC13009357; doi:10.1007/s44192-026-00392-w)
Supplement: Supplementary file 1 — Supplementary Material 1 [file 44192_2026_392_MOESM1_ESM.docx]

**Supplementary Table 1**

**Supplementary Table 1. Exploratory factor analysis of the PAS Risk Questionnaire (principal axis factoring, Promax rotation) (N = 1,503)**

| **Item** | **Short label** | **Factor 1 PV** | **Factor 2 SE** | **Factor 3 SUB** | **Factor 4 BPS** | **h²** |
| --- | --- | --- | --- | --- | --- | --- |
| PV1 | Emotional exhaustion | **.78** | .18 | .12 | .21 | .68 |
| PV2 | Difficulty relaxing | **.72** | .20 | .14 | .25 | .63 |
| PV3 | Worry about the future | **.70** | .19 | .17 | .23 | .61 |
| PV4 | Academic coping difficulty | **.75** | .16 | .14 | .28 | .66 |
| PV5 | Constant tension | **.69** | .21 | .18 | .26 | .60 |
| PV6 | Maladaptive coping | **.73** | .17 | .27 | .19 | .64 |
| SE1 | Friends use PAS | .21 | **.74** | .26 | .17 | .59 |
| SE2 | PAS availability at gatherings | .18 | **.70** | .30 | .20 | .58 |
| SE3 | Peer pressure | .22 | **.77** | .24 | .15 | .63 |
| SE4 | Easy access to PAS | .19 | **.72** | .28 | .21 | .61 |
| SE5 | PAS common among athletes | .17 | **.68** | .25 | .23 | .55 |
| SUB1 | PAS use frequency | .16 | .24 | **.71** | .19 | .55 |
| SUB2 | Use in social settings | .14 | .31 | **.75** | .16 | .60 |
| SUB3 | PAS to cope with stress | .25 | .27 | **.69** | .23 | .57 |
| SUB4 | Trying multiple PAS types | .12 | .29 | **.77** | .18 | .62 |
| SUB5 | Difficulty reducing use | .18 | .21 | **.73** | .27 | .59 |
| BPS1 | Physical exhaustion from training | .29 | .16 | .19 | **.76** | .65 |
| BPS2 | Poor recovery after training | .25 | .13 | .24 | **.78** | .67 |
| BPS3 | Academic overload | .32 | .18 | .21 | **.72** | .61 |
| BPS4 | Sleep problems | .28 | .20 | .22 | **.71** | .59 |
| BPS5 | Craving | .24 | .23 | .29 | **.69** | .56 |
| BPS6 | Accumulated stress | .34 | .19 | .20 | **.74** | .63 |

*Note.*

*Primary factor loadings are highlighted; secondary loadings < .30 are reported to demonstrate factor separation. Internal consistency estimates reported in Table 4 closely correspond to the factor structure, indicating coherent and homogeneous subscales.*

*Extraction Method: Principal Axis Factoring.*

*Rotation: Promax with Kaiser normalisation.*

*PV = Psychological Vulnerabilities; SE = Social Environment; SUB = Substance-Use Behaviours; BPS = Biopsychosocial Stressors.*

*Only primary loadings ≥ .40 are reported and interpreted.*

*Cross-loadings remained < .30, supporting a clear multi-factor structure.*

*Factor correlations were moderate in magnitude, supporting the use of an oblique rotation.*

*Although craving may conceptually overlap with substance-use tendencies, its loading pattern indicated stronger alignment with cumulative stress and fatigue indicators, supporting its classification within the biopsychosocial stressor domain.*
